# Impact of drug price adjustments on utilization of and expenditures on angiotensin-converting enzyme inhibitors and angiotensin receptor blockers in Taiwan

**DOI:** 10.1186/1471-2458-12-288

**Published:** 2012-04-20

**Authors:** Shiou-Huei Huang, Chien-Ning Hsu, Shu-Hui Yu, Thau-Ming Cham

**Affiliations:** 1 School of Pharmacy, College of Pharmacy, Kaohsiung Medical University, No. 100, Shih-Chuan 1st Road, Kaohsiung, 807, Taiwan; 2 Department of Pharmacy, Kaohsiung Chang Gung Memorial Hospital and Chang Gung University College of Medicine, No. 123, Dabi Road, Niausung Shiang, 833, Kaohsiung, Taiwan; 3 Graduate Institute of Clinical Pharmacy, College of Pharmacy, Kaohsiung Medical University, No. 100, Shih-Chuan 1st Road, Kaohsiung, 807, Taiwan; 4 Institute of Statistics, National University of Kaohsiung, No. 700, Kaohsiung University Road, Nanzih District, 811, Kaohsiung, Taiwan

**Keywords:** Drug price adjustments, Generic drug, Brand-name drug, Prescription switching, Patented drug, Off-patent drug, Drug utilization, Drug expenditures

## Abstract

**Background:**

A previous study has suggested that drug price adjustments allow physicians in Taiwan to gain greater profit by prescribing generic drugs. To better understand the effect of price adjustments on physician choice, this study used renin-angiotensin drugs (including angiotensin-converting enzyme inhibitors [ACEIs] and angiotensin receptor blockers [ARBs]) to examine the impact of price adjustments on utilization of and expenditures on patented and off-patent drugs with the same therapeutic indication.

**Methods:**

Using the Taiwan’s Longitudinal Health Insurance Database (2005), we identified 147,157 patients received ACEIs and/or ARBs between 1997 and 2008. The annual incident and prevalent users of ACEIs, ARBs and overall renin-angiotensin drugs were examined. Box-Tiao intervention analysis was applied to assess the impact of price adjustments on monthly utilization of and expenditures on these drugs. ACEIs were divided into patented and off-patent drugs, off-patent ACEIs were further divided into original brands and generics, and subgroup analyses were performed.

**Results:**

The number of incident renin-angiotensin drug users decreased over the study period. The number of prevalent ARB users increased and exceeded the cumulative number of first-time renin-angiotensin drug users starting on ARBs, implying that some patients switched from ACEIs to ARBs. After price adjustments, long term trend increases in utilization were observed for patented ACEIs and ARBs; a long-term trend decrease was observed for off-patent ACEIs; long-term trend change was not significant for overall renin-angiotensin drugs. Significant long-term trend increases in expenditures were observed for patented ACEIs after price adjustment in 2007 (200.9%, p = 0.0088) and in ARBs after price adjustments in 2001 (173.4%, p < 0.0001) and 2007 (146.3%, p < 0.0001). A significant long-term trend decrease in expenditures was observed for off-patent ACEIs after 2004 price adjustment (−156.9%, p < 0.0001). Expenditures on overall renin-angiotensin drugs showed long-term trend increases after price adjustments in 2001 (72.2%, p < 0.0001) and 2007 (133.4%, p < 0.0001).

**Conclusions:**

Price adjustments did not achieve long-term cost savings for overall renin-angiotensin drugs. Possible switching from ACEIs to ARBs within individuals is evident. Policy makers should reconsider the appropriateness of the current adjustment strategies applied to patented and off-patent drugs.

## Background

Taiwan’s National Health Insurance (NHI) system is a government-run, single-payer, compulsory program implemented on March 1, 1995. The Bureau of National Health Insurance (BNHI) is the executive organization of the NHI program. This program has universal coverage, including pharmaceuticals, ambulatory care, inpatient care, traditional Chinese medicine, dental services, child delivery, rehabilitation, home nursing care and chronic psychiatric rehabilitation. Currently, it covers more than 99% of the population (approximately 23 million people) [1].

The three main components of the NHI system are the BNHI, the insured and the BNHI-contracted health care providers. Funding for the BNHI comes from the insured (38%), the employers (36%) and the government (26%). The BNHI issues insurance cards to the insured and pays the health care providers according to the services they provide, including prescriptions listed in the Pharmaceutical Benefit scheme (PBS). When the insured receive medical services from BNHI-contracted health care providers, they only pay the provider a registration fee as well as any co-payment for outpatient services, inpatient services and drugs [1].

**Figure 1:**
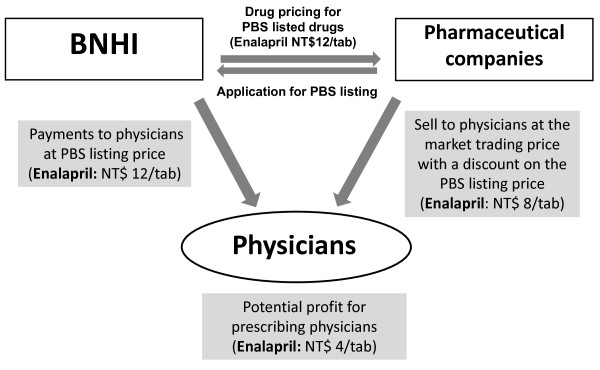
**PBS reimbursement price vs. market trading price: an example of a generic ACEI ─ Enalapril.** ACEIs, angiotensin-converting enzyme inhibitors; BNHI, Bureau of National Health Insurance; NT$, New Taiwan Dollars (at an exchange rate of NT$30.39 to US$1 on June 30, 2008); PBS, Pharmaceutical Benefit Scheme.

The BNHI established the PBS in 1996. It contains the reimbursement principles, and a list of the reimbursed products with a brand-specific reimbursement price for each product. This reimbursement price is paid to physicians, as they are responsible for purchasing, prescribing and dispensing in Taiwan [2]. However, physicians can purchase medications from pharmaceutical companies at the market trading price with a discount on the PBS reimbursement price. A profit margin for physicians then develops because of the difference between the reimbursement price and the market trading price; this is known as drug price deviation. Figure 1 provides an example of this pricing and payment structure. Under this reimbursement structure, physicians’ prescribing decisions have been hypothesized to be influenced by the drug price deviation [3].

The NHI, like many health insurance programs, faces financial challenges and its expenditures have exceeded its revenue since 1998 [3]. Annual pharmaceutical expenditures have doubled from 62.2 billion New Taiwan Dollars (NT$; at an exchange rate of NT$30.39 to US$1 on June 30, 2008) in 1996 to NT$131.3 billion in 2010 [1], and the BNHI is under pressure to control outlays. Stepwise price adjustments (PA), which aim to reduce the reimbursement price to be close to the market trading price, is the major cost containment strategy adopted by the BNHI since 1996 [4]. In the early stage of price adjustments (1996–1997) reimbursement price adjustments were based on the prices of international products or inter-brand comparison of existing products. On April 1, 2000 (PA2000), the BNHI began to conduct market price and volume surveys before each price adjustment, and adjusted the reimbursement price of each individual drug according to its weighted average market trading price (WAP) calculated from the market survey. Above the WAP, the BNHI allows a reasonable profit margin (known as the reasonable zone or “r-zone”) for physicians. The “r-zone” for patented drugs has been higher than that for off-patent drugs since November 1, 2006 (PA2006). Since April 1, 2001 (PA2001), the BNHI has adopted different strategies for patented and off-patent drugs. The reimbursement prices of patented drugs are still adjusted based on the individual drug’s WAP, but those of off-patent drugs are adjusted according to the principle of “generic grouping”. Under this principle, drugs with the same active ingredients, content, and strength are grouped together; and their reimbursement prices are adjusted according to a group weighted average price (GWAP) calculated from their market trading prices. The Additional file 1 provides the details of the PBS and an example to illustrate the pricing structure.

Price adjustments based on the market price and volume survey and generic grouping have, from the BNHI’s perspective, demonstrated cost savings in pharmaceutical expenditures for all PBS listed drugs [5]. Liu et al. [6] concluded that the financial incentive offered by the drug price deviation is the major driving force for physicians to switch from a branded drug to its generic version. However, previous studies did not investigate the potential switching between patented and off-patent drugs after price adjustments. It is critical to analyze whether price adjustments result in prescription switching between these products, and whether that eventually leads to the anticipated cost savings without compromising patient safety and therapeutic effectiveness.

ACEIs (angiotensin-converting enzyme inhibitors) and ARBs (angiotensin receptor blockers) both work through the same renin-angiotensin pathway and have a similar effect in cardiovascular disease and renal protection [7]. Because several ACEIs were launched in Taiwan before 1997, and their off-patent products heavily dominated the market share over time, they provide an ideal example for obtaining knowledge about the longitudinal changes in medication utilization and expenditures under stepwise price adjustments. Furthermore, because ARBs are generally more expensive than ACEIs, but are therapeutically similar to ACEIs, we are able to compare the impact of price adjustments on the two classes. Therefore, this study uses ACEIs and ARBs as examples to examine the impact of Taiwan’s stepwise price adjustments on utilization of and expenditures on patented and off-patent drugs.

## Methods

### Study design and data source

This retrospective, observational study was based on a cohort of patients treated with ACEIs and/or ARBs within Taiwan’s NHI program. The Longitudinal Health Insurance Database 2005 (LHID2005) [8] contains all the original claim data of one million beneficiaries randomly sampled from the year 2005 Registry for Beneficiaries of the National Health Insurance Research Database (NHIRD), which contains registration data of everyone who was a beneficiary of the NHI program during the period of January 1, 2005, to January 1, 2006. The BNHI and the National Health Research Institutes (NHRI) provided access to the LHID2005 (registered numbers: 98177 and 98251). Among the one million randomly sampled individuals, clinical conditions were identified based on the International Classification of Disease Version 9 Clinical Modification (ICD9-CM) codes and the A-codes used initially in Taiwan’s NHI program [9]. Utilization of and expenditures on drugs were determined for inpatient and outpatient services at all BNHI-contracted health settings and pharmacies.

### Patient selection

Patients with at least one ATC code [10] for ACEIs (from January 1997 to December 2008) or ARBs (from February 1998 to December 2008) were included and were regarded as the whole sample in this study, defined as renin-angiotensin drug users. These patients were further classified into three groups: ACEI users (only used ACEIs during the study period), ARB users (only used ARBs during the study period), and both drugs users (used at least one ACEI and one ARB concurrently or subsequently during the study period). For each year, from 1997 through 2008, the prevalent and incident users of ACEIs, ARBs, both drugs (ACEIs and ARBs used concurrently or subsequently in a given year) and overall renin-angiotensin drugs (ACEIs and ARBs used alone or in combination in a given year) were calculated. ACEIs can be used as an example to explain the meaning of incident and prevalent users. Incident ACEI users in a given year were defined as patients who received ACEIs for the first time in that year and who did not have drug claims for ACEIs and ARBs alone or in combination in the preceding years. Prevalent ACEI users in a given year were defined as patients who received ACEIs in that year. The date of birth, gender and clinical conditions of the patients were retrieved for the analyses. Age (in years) was determined at the earliest date of ACEIs or ARBs prescription. The presence of cardiovascular disease (CVD, ICD9-CM, 390–459), diabetes (ICD9-CM, 249–250), kidney disease (ICD9-CM, 580–589), and hyperlipidemia (ICD9-CM, 272) [11] was determined by patients having at least one ICD9-CM code for the individual diseases.

### Drug utilization, expenditures and price adjustment policy

Defined daily dose (DDD) [10] was used to represent the utilization of the investigated agents. Expenditures were expressed in NT$. The utilization of and expenditures on ACEIs and ARBs were expressed in months to show longitudinal changes over time.

Seven drug price adjustments were examined, including one based on the market price and volume survey implemented on April 1, 2000 (PA2000) and six based on the market survey in combination with generic grouping implemented on April 1, 2001 (PA2001), March 1, 2003 (PA2003), November 1, 2004 (PA2004), September 1, 2005 (PA2005), November 1, 2006 (PA2006) and September 1, 2007 (PA2007).

### Statistical analyses

Multivariate logistic regression was used to explore the differences between ACEI users, ARB users and both drugs users. Odds ratios (OR) and 95% confidence intervals (95% CI) were calculated.

Box-Tiao intervention analysis [12,13] was applied to determine whether each price adjustment was associated with significant changes in utilization of and expenditures on ACEIs, ARBs and overall renin-angiotensin drugs, after controlling for potential confounding factors. In the intervention analysis, we fitted the independent variables first and then applied the autoregressive integrated moving-average (ARIMA) modeling identification process to the residuals.

Therefore, we considered the following model [13,14],

(1) $$y_{t}=\beta_{0}+\beta_{1}t+\overset{n}{\underset{i=1}{\Sigma }}\left(\beta_{2i}PA_{\text{it}}+\beta_{2i+1}TPA_{\mathrm{it}}\right)+\frac{\theta \left(B\right)}{\phi \left(B\right)}a_{t}\text{,}\text{for}t=1,\ldots ,T$$

where *y*_*t*_ is the dependent variable (i.e., response series), *t* is the baseline trend, denoting months in numerical order, from 1 to *T* (*T* is the sample size), and *n* is the number of price adjustments in this study. *PA*_*it*_ is a level indicator function to indicate whether the level changed as the *i*th price adjustment occurred. That is, assuming the *i*th price adjustment occurred on $t=t_{0}$, let *PA*_*it*_ be 0 for $t<t_{0}$, and 1 for $t\geq t_{0}$. *TPA*_*it*_ is a trend variable to indicate whether the trend changed as the *i*th price adjustment occurred, that is, let *TPA*_*it*_ be 0 for $t<t_{0}$, and $t-t_{0}$+1, for $t\geq t_{0}$. *β*_*0*_ is the constant term that describes the baseline level of the dependent variable. *β*_*1*_ describes the baseline trend before the first intervention occurred. *β*_*2i*_ depicts the level change immediately after the *i*th price adjustment and *β*_*2i+1*_ depicts the trend change after the *i*th price adjustment (i.e., it compares the monthly trend after the *i*th price adjustment with the monthly trend before the *i*th price adjustment). *B* is the backshift operator (i.e.,$B^{i}X_{t}=X_{t-i}$). $\phi \left(B\right)=1-\phi_{1}B-\cdots -\phi_{p}B^{p}$is the autoregressive polynomial of the model, and $\theta \left(B\right)=1-\theta_{1}B-\cdots -\theta_{q}B^{q}$ is the moving average polynomial. *a*_*t*_ is the white noise with mean 0 and variance σ^2^.

Dependent variables in primary analyses were the monthly utilization of and expenditures on ACEIs, ARBs and overall renin-angiotensin drugs. In subgroup analyses, the monthly utilization of and expenditures on ACEIs were categorized into patented drugs (benazepril, cilazapril, imidapril, perindopril and ramipril) and off-patent drugs (captopril, off-patent from PA2003; enalapril, fosinopril, lisinopril and quinapril, off-patent from PA2006). The off-patent ACEIs were further categorized into original branded and generic versions. Potential independent variables included a constant (baseline level); a baseline trend; two indicator variables for each price adjustment, namely level change (the immediate effect) and trend change (the long-term effect or changes over time) [14]; and three confounding factors. One of the three confounding factors was the global budget system implemented in Taiwan’s hospitals on July 1, 2002 (GB2002) that increased outpatient use of anti-diabetic and anti-hypertensive agents [15], and increased expenditures on all PBS listed drugs [5]. The other two confounding factors were the Chinese New Year (CNY) [5,16] and the outbreak of severe acute respiratory syndrome (SARS) in 2003 [5], both of which decreased expenditures on all PBS listed drugs. When modeling each dependent variable, we removed a number of potential independent variables representing the cut-points of price adjustments, and only selected some of them to incorporate into the model (a so-called parsimonious model). These independent variables were initially selected using a backward elimination procedure. Collinearity diagnostics were subsequently performed to remove variables until the condition index was less than 30 [13,17]. ARIMA modeling expressed in factored form [13] was applied to the residuals. Several candidate models were considered according to the autocorrelation plots and partial autocorrelation plots. The model with the minimum Akaike Information Criterion (AIC) value was chosen as the best fit. The Ljung-Box chi-square statistic revealed insignificant autocorrelation for the residuals [13]. All statistical analyses were performed using SAS 9.1 (SAS, Cary, NC) with a p-value of 0.05 considered significant.

## Results

### Patients

Of the 147 157 patients who met the inclusion criteria, 64 710, 22 317and 60 130 were identified as ACEI users, ARB users and both drugs users, respectively (Table 1). Among these patients, those aged under 60 years were less likely to be ARB users and both drugs users than ACEI users. Male patients were more likely to be ARB users than ACEI users (OR = 1.08, 95% CI: 1.04–1.11), but they were less likely to be both drugs users than ACEI users (OR = 0.91, 95% CI: 0.89–0.93). Patients with hyperlipidemia (OR = 1.70, 95% CI: 1.64–1.77) were more likely to take ARBs than ACEIs. Patients at risk of CVD (OR = 6.14, 95% CI: 5.54–6.81), diabetes (OR = 2.04, 95% CI: 1.98–2.10), kidney disease (OR = 2.96, 95% CI: 2.83–3.10), and hyperlipidemia (OR = 2.63, 95% CI: 2.56–2.71) revealed a higher likelihood of being prescribed both drugs rather than just ACEIs.

**Table 1:** Characteristics of ACEI users, ARB users and both drugs users

Characteristics^*^	ACEI users (n = 64,710)		ARB users (n = 22,317)		Both drug users (n = 60,130)		ARB users vs. ACEI users^§^ Adjusted OR (95%CI)		Both drug users vs. ACEI users^§^ Adjusted OR (95%CI)	
**Age**^**†**^**, mean (SD)**	54.7	(17.1)	57.8	(14.0)	59.7	(12.8)				
< 20 yrs	2,657	(4.1)	71	(0.3)	102	(0.2)	0.13	(0.10–0.17)^#^	0.19	(0.15–0.23)^#^
20–39 yrs	7,197	(11.1)	1,910	(8.6)	3,532	(5.9)	0.81	(0.77–0.86)^#^	0.60	(0.57–0.63)^¶^
40–59 yrs	28,172	(43.5)	10,534	(47.2)	24,565	(40.9)	0.99	(0.96–1.02)^#^	0.72	(0.71–0.74)^#^
> = 60 yrs (reference group)	26,684	(41.2)	9,802	(43.9)	31,931	(53.1)		1		1
**Sex**										
Male	33,267	(51.4)	11,984	(53.7)	29,810	(49.6)	1.08	(1.04–1.11)^#^	0.91	(0.89–0.93)^#^
Female (reference group)	31,433	(48.6)	10,333	(46.3)	30,320	(50.4)		1		1
**Clinical conditions**^**‡**^										
Cardiovascular disease (CVD) risk (390–459)	55,253	(85.4)	20,526	(92.0)	59,489	(98.9)	0.63	(0.57–0.68)^#^	6.14	(5.54–6.81)^#^
Diabetes (249–250)	11,595	(17.9)	4,837	(21.7)	24,311	(40.4)	0.93	(0.90–0.97)^¶^	2.04	(1.98–2.10)^#^
Kidney diseases (580–589)	3,493	(5.4)	1,215	(5.4)	9,863	(16.4)	0.84	(0.79–0.91)^#^	2.96	(2.83–3.10)^#^
Hyperlipidemia (272)	11,247	(17.4)	6,353	(28.5)	26,004	(43.2)	1.70	(1.64–1.77)^#^	2.63	(2.56–2.71)^#^
None of the above diseases	7,745	(12.0)	841	(3.8)	113	(0.2)	0.28	(0.25–0.31)^#^	0.21	(0.17–0.26)^#^

^*^Unless otherwise indicated, values are numbers and proportions (in %).

^†^Age is defined at the earliest date of ACEIs or ARBs prescription.

^‡^The clinical conditions were represented by ICD9-CM codes.

^§^The adjusted odds ratios were estimated in multivariate logistic regression with the reference group defined as patients who were treated only with ACEIs during the study period. Patients who were treated only with ARBs were defined as ARB users. Patients who were treated with at least one ACEI and one ARB during the study period were defined as both drug users.

^¶^Significance is represented as: p < 0.01, ^#^p < 0.0001.

ACEI, angiotensin-converting enzyme inhibitor; ARB, angiotensin receptor blocker; OR, odds ratio; SD, standard deviation.

**Figure 2:**
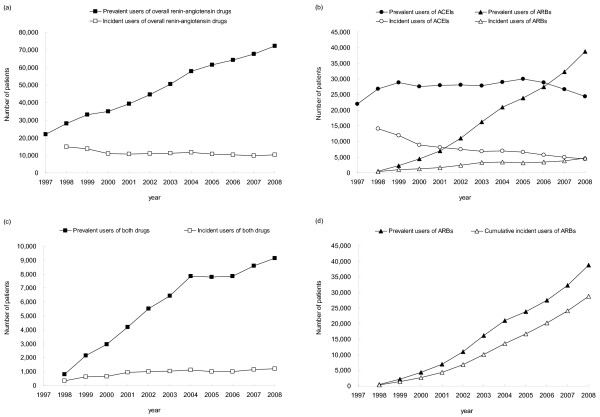
**The number of annual incident and prevalent users for ACEIs and ARBs.** (**a**) Annual incident and prevalent users of renin-angiotensin drugs (ACEIs and/or ARBs); (**b**) Annual incident and prevalent users of ACEIs or ARBs; (**c**) Annual incident and prevalent users of both drugs (i.e., ACEIs and ARBs used concurrently or subsequently); (**d**) Annual prevalent ARB users and cumulative incident ARB users. ACEIs, angiotensin-converting enzyme inhibitors; ARBs, angiotensin receptor blockers.

Figure 2a–2c depict the annual prevalent (from 1997 to 2008) and incident (from 1998 to 2008) users of overall renin-angiotensin drugs, ACEIs, ARBs, and both drugs. For renin-angiotensin drugs, the number of incident users decreased from 14 848 to 10 325 (% change: -30.5%) from 1998 to 2008, while the number of prevalent users increased from 22 049 to 72 278 (227.8%) from 1997 to 2008 (Figure 2a). For ACEIs, the number of incident users declined from 14 104 to 4 420 (−68.7%) from 1998 to 2008, whilst the number of prevalent users increased from 22 049 to 29 949 (35.8%) from 1997 to 2005 and declined from 29 949 to 24 369 (−18.6%) from 2005 to 2008. For ARBs, the number of incident users increased from 399 to 4 704 (1078.9%), and the number of prevalent users increased from 498 to 38 758 (7682.7%) from 1998 to 2008 (Figure 2b). For users of both drugs, the number of incident users increased from 345 to 1 201 (248.1%) and the number of prevalent users increased from 811 to 9 151 (1028.4%) from 1998 to 2008 (Figure 2c). It is noteworthy that the number of prevalent ARB users increased rapidly between 1998 and 2008, and has exceeded the number of prevalent ACEI users since 2007. However, the number of incident ARB users only increased slightly year by year (Figure 2b), and the number of cumulative incident ARB users (from 1998 to a given year) was always less than the number of prevalent ARB users in each year, with the former only being 60 to 80% of the latter (Figure 2d). This indicates that annual prevalent ARB users were not only from the category of cumulative incident ARB users, but also included users who initially used ACEIs, or both drugs, in preceding years and switched to ARBs thereafter.

### Drug utilization

**Figure 3:**
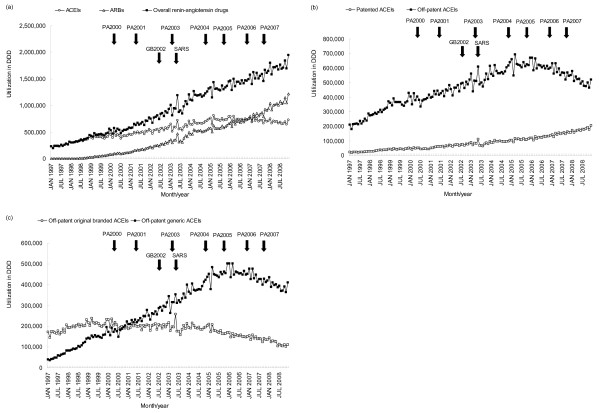
**Monthly utilization of ACEIs, ARBs, and overall renin-angiotensin drugs.** (**a**) ACEIs, ARBs and renin-angiotensin drugs; (**b**) patented and off-patent ACEIs; (**c**) original branded and generic off-patent ACEIs. The arrows indicate the time points at which price adjustments were implemented. ACEIs, angiotensin-converting enzyme inhibitors; ARBs, angiotensin receptor blockers; DDD, defined daily dose; PA2000, price adjustment implemented on April 1, 2000; PA2001, price adjustment implemented on April 1, 2001; PA2003, price adjustment implemented on March 1, 2003; PA2004, price adjustment implemented on November 1, 2004; PA2005, price adjustment implemented on September 1, 2005; PA2006, price adjustment implemented on November 1, 2006; PA2007, price adjustment implemented on September 1, 2007.

The baseline level of utilization of ACEIs was 232 380 DDD and increased by 5 531 DDD (*p* < 0.0001) per month from January 1997. There was an immediate decrease of 48 286 DDD (level change: -20.8%, *p* < 0.0001) when PA2000 was implemented, but an immediate increase of 28 987 DDD (level change: 15.7%, *p* = 0.0073) following PA2004. ACEIs long-term increasing trend decreased to 671 DDD per month after the implementation of PA2004 (trend change: -87.9%, *p* < 0.0001) and turned to a long-term decreasing trend of 2 858 DDD per month after the implementation of PA2006 (trend change: -525.9%, *p* = 0.0002), diminishing to 691 742 DDD in December 2008 (Figure 3a and Table 2). In subgroup analyses, we observed that the utilization of off-patent ACEIs showed a similar trend to that of ACEIs, but the baseline increasing trend turned to a downward trend even earlier following PA2004. On the other hand, the utilization of patented ACEIs showed an increasing trend throughout the study period (Figure 3b and Table 2). When off-patent ACEIs were further categorized into original branded and generic versions, we found that the baseline increasing trend for the utilization of original branded version turned to a decreasing trend after the implementation of PA2004. Generic versions still showed an upward trend at PA2004 and only turned to a downward trend afterward when PA2005 was implemented (Figure 3c and Table 2).

**Table 2:** Impact of price adjustments on monthly utilization of ACEIs, ARBs and overall renin-angiotensin drugs

Dependent variables	Time period	Factored ARIMA model^*^	Independent variables^†^	Coefficients^‡^	*p* value
**Primary analyses**					
ACEIs	Jan 1997 to Dec 2008	p = (6);	Constant	232,380	<0.0001
		q = (6)(12)	Baseline trend	5,531	<0.0001
			PA2000 level change	-48,286	<0.0001
			PA2004 level change	28,987	0.0073
			PA2004 trend change	-4,860	<0.0001
			PA2006 trend change	-3,529	0.0002
			CNY	-36,256	<0.0001
ARB	Feb 1998 to Dec 2008	p = (2,3)(12)	Baseline trend	5,044	<0.0001
			PA2003 trend change	5,014	<0.0001
			PA2007 trend change	8,583	0.0003
			CNY	-21,476	0.0219
Renin-angiotensin drugs	Jan 1997 to Dec 2008	p = (2,5)(12)	Constant	135,289	<0.0001
			Baseline trend	12,027	<0.0001
			PA2000 level change	-73,794	0.0053
			CNY	-32,789	0.0371
**Subgroup analyses for ACEIs**					
Patented ACEIs	Jan 1997 to Dec 2008	p = (6);	Constant	18,039	<0.0001
		q = (6)	Baseline trend	872	<0.0001
			PA2000 level change	-9,466	<0.0001
			PA2001 level change	6,416	0.0022
			PA2004 trend change	1,235	<0.0001
			PA2005 level change	-13,602	<0.0001
			CNY	-7,318	<0.0001
Off-patent ACEIs	Jan 1997 to Dec 2008	p = (12);	Constant	209,374	<0.0001
		q = (9)	Baseline trend	4,617	<0.0001
			PA2000 level change	-37,219	<0.0001
			PA2004 level change	26,145	0.0028
			PA2004 trend change	-5,201	<0.0001
			PA2006 trend change	-4,593	<0.0001
			CNY	-20,665	0.0021
Off-patent original branded ACEIs	Jan 1997 to Dec2008	p = (1,4,5)(12)	Constant	179,478	<0.0001
			Baseline trend	527	0.0081
			PA2000 level change	-21,380	0.0031
			PA2004 trend change	-2,574	<0.0001
			CNY	-11,501	0.0003
Off-patent generic ACEIs	Jan 1997 to Dec 2008	p = (12);	Constant	25,416	<0.0001
		q = (12)	Baseline trend	4,205	<0.0001
			PA2000 level change	-23,138	<0.0001
			PA2004 level change	22,925	<0.0001
			PA2005 trend change	-4,315	<0.0001
			PA2006 trend change	-3,197	<0.0001
			CNY	-9,447	0.0445

^*^Factored ARIMA model is represented by p, d, q: p, auto-regressive order; d, differencing order; q, moving-average order; seasonal order is represented by p = (12), d = (12) or q = (12). Take ACEIs as an example. The moving-average operator represented by q = (6)(12) means

(*1-θ*_*6*_*B*^*6*^*)* (*1-θ*_*12*_*B*^*12*^*).*

^†^Parsimonious models were adopted, and therefore, only significant independent variables were incorporated into the model.

^‡^The unit of the regression coefficient is defined daily dose (DDD).

ACEIs, angiotensin-converting enzyme inhibitors; ARBs, angiotensin receptor blocker; ARIMA, auto-regressive integrated moving-average;

CNY, Chinese new year; PA2000, price adjustment implemented on April 1, 2000; PA2001, price adjustment implemented on April 1, 2001; PA2003, price adjustment implemented on March 1, 2003; PA2004, price adjustment implemented on November 1, 2004; PA2005, price adjustment implemented on September 1, 2005; PA2006, price adjustment implemented on November 1, 2006; PA2007, price adjustment implemented on September 1, 2007.

Unlike ACEIs, a long-term increasing trend in the utilization of ARBs and overall renin-angiotensin drugs was observed from 1998 to 2008 and from 1997 to 2008, respectively. The baseline level of the utilization of ARBs was zero and increased by 5 044 DDD (*p* < 0.0001) per month from February 1998. Following PA2003, the long-term increasing trend of the use of ARBs further increased to 10 058 DDD per month (trend change: 99.4%, *p* < 0.0001). When PA2007 was implemented, ARBs long-term increasing trend further increased to 18 041 DDD per month (trend change: 85.3%, *p* = 0.0003), reaching 1 157 724 DDD in December 2008. The baseline level of the utilization of overall renin-angiotensin drugs was 135 289 DDD, and utilization showed a long-term increasing trend of 12 027 DDD (*p* < 0.0001) per month from January 1997. When PA2000 was implemented, overall usage experienced an immediate decrease of 73 794 DDD (level change: -54.5%, *p* = 0.0053). However, throughout the study period, the long-term increasing trend in the utilization of overall renin-angiotensin drugs was not influenced by any price adjustment, reaching 1 817 084 DDD in December 2008 (Figure 3a and Table 2).

With regard to the three confounding factors, only CNY significantly resulted in immediate decreases in the utilization of ACEIs (including in subgroup analyses), ARBs and overall renin-angiotensin drugs (Table 2).

### Drug expenditures

**Figure 4:**
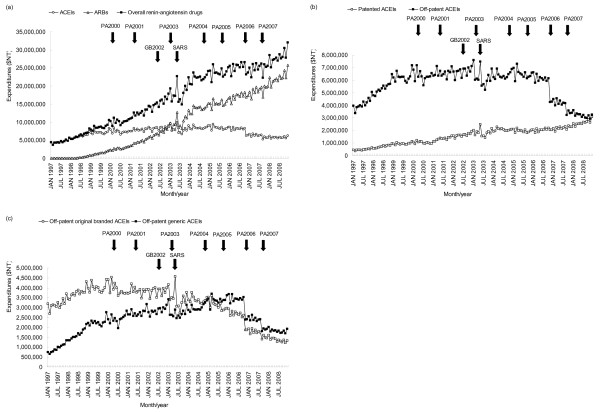
**Monthly expenditures on ACEIs, ARBs, and overall renin-angiotensin drugs.** (**a**) ACEIs, ARBs and renin-angiotensin drugs; (**b**) patented and off-patent ACEIs; (**c**) original branded and generic off-patent ACEIs. The arrows indicate the time points at which price adjustments were implemented. ACEIs, angiotensin-converting enzyme inhibitors; ARBs, angiotensin receptor blockers; NT$, New Taiwan Dollars (at an exchange rate of NT$30.39 to US$1 on June 30, 2008); PA2000, price adjustment implemented on April 1, 2000; PA2001, price adjustment implemented on April 1, 2001; PA2003, price adjustment implemented on March 1, 2003; PA2004, price adjustment implemented on November 1, 2004; PA2005, price adjustment implemented on September 1, 2005; PA2006, price adjustment implemented on November 1, 2006; PA2007, price adjustment implemented on September 1, 2007.

The baseline level of expenditures on ACEIs was 4 509 908 NT$ and increased by 66 568 NT$ (*p* < 0.0001) per month from January 1997. The expenditures on ACEIs experienced an immediate decrease of 592 252 NT$ (level change: -13.1%, *p* = 0.0151) and 1 255 287 NT$ (level change: -32.0%, *p* < 0.0001), respectively, following PA2000 and PA2003. The long-term increasing trend in ACEI expenditures turned to a downward trend of 30 014 NT$ per month after the implementation of PA2004 (trend change: -145.1%, *p* < 0.0001). When PA2006 was implemented, ACEI expenditures showed an immediate decrease of 1 545 767 NT$ (level change: -58.1%, *p* < 0.0001). After PA2006, it maintained the long-term downward trend of 30 014 NT$ per month, decreasing to 5 527 281 NT$ in December 2008 (Figure 4a and Table 3). In subgroup analyses, we observed that expenditures on off-patent ACEIs showed a similar trend to that of ACEIs overall. The baseline increasing trend also turned to a decreasing trend following PA2004, but experienced an additional level reduction following PA2007. On the other hand, expenditures on patented ACEIs showed an increasing trend throughout the study period (Figure 4b and Table 3). When off-patent ACEIs were further categorized into original branded and generic versions, we found that expenditures on off-patent original branded ACEIs showed a similar trend to off-patent ACEIs. Expenditures on off-patent generic ACEIs only showed an immediate decrease when PA2003, PA2006 and PA2007 were implemented, but was not influenced by any price adjustment in the long term (Figure 4c and Table 3).

**Table 3:** Impacts of price adjustments on monthly expenditures on ACEIs, ARBs and overall renin-angiotensin drugs

Dependent variables	Time period	Factored ARIMA model^*^	Independent variables^†^	Coefficients^‡^	*p* value
**Primary analyses**					
ACEIs	Jan 1997 toDec 2008	p = (1)(12);	Constant	4,509,908	<0.0001
		q = (1)(9)	Baseline trend	66,568	<0.0001
			PA2000 level change	-592,252	0.0151
			PA2003 level change	-1,255,287	<0.0001
			PA2004 trend change	-96,582	<0.0001
			PA2006 level change	-1,545,767	<0.0001
			CNY	-351,216	0.0029
ARB	Feb 1998 toDec 2008	p = (12);	Baseline trend	92,537	<0.0001
		q = (2)	PA2001 trend change	160,472	<0.0001
			PA2004 trend change	-120,338	<0.0001
			PA2007 trend change	194,043	<0.0001
			CNY	-577,052	0.0220
Renin-angiotensin drugs	Jan 1997 to Dec 2008	p = (6);	Constant	3,753,898	<0.0001
		q = (6)	Baseline trend	152,800	<0.0001
			PA2001 trend change	110,259	<0.0001
			PA2004 trend change	-133,325	<0.0001
			PA2006 level change	-2,224,884	<0.0001
			PA2007 trend change	173,056	<0.0001
			CNY	-1,108,357	0.0011
**Subgroup analyses for ACEIs**					
Patented ACEIs	Jan 1997 to Dec 2008	p = (1)(6,12);	Constant	497,488	<0.0001
		q = (1)	Baseline trend	12,684	<0.0001
			PA2001 level change	189,675	0.0281
			PA2007 trend change	25,478	0.0088
			CNY	-116,498	0.0011
Off-patent ACEIs	Jan 1997 to Dec 2008	p = (1)(12);	Constant	3,987,107	<0.0001
		q = (1)(9)	Baseline trend	45,221	<0.0001
			PA2000 level change	-380,037	0.0251
			PA2003 level change	-1,022,868	<0.0001
			PA2004 trend change	-70,970	<0.0001
			PA2006 level change	-1,675,618	<0.0001
			PA2007 level change	-689,097	0.0002
			CNY	-239,858	0.0034
Off-patent original branded ACEIs	Jan 1997 to Dec 2008	p = (1)(12);	Constant	3,195,426	<0.0001
		q = (1)(9)	Baseline trend	12,121	0.0081
			PA2000 level change	-266,177	0.0271
			PA2003 level change	-498,038	<0.0001
			PA2004 trend change	-46,182	<0.0001
			PA2006 level change	-575,144	<0.0001
			CNY	-182,498	0.0019
Off-patent generic ACEIs	Jan 1997 to Dec 2008	p = (1)(12);	Constant	985,881	0.0269
		q = (1)(5,9)	Baseline trend	27,153	0.0008
			PA2003 level change	-525,353	<0.0001
			PA2006 level change	-1,092,328	<0.0001
			PA2007 level change	-524,951	<0.0001

^*^Factored ARIMA model is represented by p, d, q: p, auto-regressive order; d, differencing order; q, moving-average order; seasonal order is represented by p = (12), d = (12) or q = (12). Take patented ACEIs as an example. The autoregressive operator represented by p = (1)(6,12) means

(*1-θ*_*1*_*B)* (*1-θ*_*6*_*B*^*6*^*-θ*_*12*_*B*^*12*^*).*

^†^Parsimonious models were adopted, and therefore, only significant independent variables were incorporated into the model.

^‡^The unit of the regression coefficient is NT$ (NT$, New Taiwan Dollars; at an exchange rate of NT$30.39 to US$1 on June 30, 2008)

ACEIs, angiotensin-converting enzyme inhibitors; ARBs, angiotensin receptor blocker; ARIMA, auto-regressive integrated moving-average;

CNY, Chinese new year; PA2000, price adjustment implemented on April 1, 2000; PA2001, price adjustment implemented on April 1, 2001; PA2003, price adjustment implemented on March 1, 2003; PA2004, price adjustment implemented on November 1, 2004; PA2005, price adjustment implemented on September 1, 2005; PA2006, price adjustment implemented on November 1, 2006; PA2007, price adjustment implemented on September 1, 2007.

Unlike ACEIs, expenditures on ARBs revealed a long-term increasing trend from 1998 to 2008. The baseline level of expenditures on ARBs was zero and increased by a trend of 92 537 NT$ (*p* < 0.0001) per month from February 1998. Following PA2001, the long-term increasing trend of expenditures on ARBs increased to 253 009 NT$ per month (trend change: 173.4%, *p* < 0.0001). When PA2004 was implemented, the long-term increasing trend decreased to 132 671 NT$ per month (trend change: -47.6%, *p* = 0.0003), but following PA2007, the long-term upward trend further increased to 326 714 NT$ (trend change: 146.3%, *p* < 0.0001), reaching 24 228 254 NT$ in December 2008. The baseline level of expenditures on overall renin-angiotensin drugs was 3 753 898 NT$ and increased with a trend of 152 800 NT$ (*p* < 0.0001) per month from January 1997. When PA2001 was implemented, the long-term increasing trend increased to 263 059 NT$ per month (trend change: 72.2%, *p* < 0.0001), but following PA2004, the long-term increasing trend decreased to 129 734 NT$ per month (trend change: -50.7%, *p* < 0.0001). When PA2006 was implemented, the expenditures on overall renin-angiotensin drugs showed an immediate decrease of 2 224 884 (level change: -59.3%, *p* < 0.0001). Expenditures still maintained a long-term increasing trend of 129 734 NT$ per month until the implementation of PA2007; this upward trend further increased to 302 790 NT$ per month (trend change: 133.4%, *p* < 0.0001), reaching 30 201 508 NT$ in December 2008 (Figure 4a and Table 3).

With regard to the three confounding factors, only CNY significantly resulted in immediate decreases in the expenditures on ACEIs (except for off-patent generic ACEIs), ARBs and overall renin-angiotensin drugs (Table 3).

## Discussion

There are two major findings in our study. One is that the increase in prevalent ARB users is associated with switching from being an ACEI user. This result is consistent with the long-term trend increases in utilization of ARBs, but a long-term trend decrease in utilization of ACEIs. The other is that the increase in expenditures on renin-angiotensin drugs throughout this study primarily resulted from increases in the number of incident ARB users and potential switching from ACEIs to ARBs, but not from changes in the use of overall renin-angiotensin drugs. Ultimately, cost savings have not been achieved in overall use of renin-angiotensin drugs. Our findings differ from those of previous studies that showed cost savings in pharmaceutical expenditures for all PBS listed drugs [5] and generic substitution driven by the financial incentive for physicians [6] after price adjustments.

As mentioned, a feature of Taiwan’s health care system is that physicians both prescribe and dispense drugs because they are permitted by Taiwan’s Department of Health to hire pharmacists to work at their on-site pharmacies [2]. Physicians stand to profit from the gap between the reimbursement price and the market trading price. In 2009, Liu et al. [6] conducted a study in Taiwan to investigate prescribing preferences between original branded and generic drugs in relation to this potential profit margin, and found that prescribing of generics increased as the reimbursement price decreased. Our results were only consistent with these findings when we examined the change in utilization of off-patent ACEIs. We observed that the utilization of off-patent original branded ACEIs was lower than that of off-patent generic ACEIs since 2001. Although the initial upward trend in the use of off-patent generic ACEIs turned to a decreasing trend after the implementation of PA2005, the use of these drugs is still higher than that of off-patent original branded drugs. However, our findings differ to those of Liu et al. when looking at both ACEIs and ARBs. Since these two classes of drugs work through the same renin-angiotensin pathway and have a similar effect in cardiovascular disease and renal protection [7], physicians make a prescribing decision between ACEIs and ARBs when the patient needs a renin-angiotensin drugs. We observed that, following PA2006, the initial rising trend of the use of ACEIs turned to a decreasing trend, primarily due to the decreases in the use of off-patent ACEIs. The initial rising trend of the use of ARBs further increased following PA2003 and PA2007, and the use of ARBs exceeded that of ACEIs from 2007. In fact, the profit margin from patented and off-patent drugs varies over time, depending on when a drug turned from patented (applying WAP adjustment) to off-patent (applying GWAP adjustment) as well as the value of the r-zone (the accessible profit margin for physicians) imposed by the BNHI. Because the extent of WAP adjustment for patented drugs is smaller than that of the GWAP adjustment for off-patent drugs, and because the r-zone is larger for patented drugs than for off-patent drugs, patented drugs offer higher financial incentive to physicians through stepwise price adjustments.

Another study conducted by Lee et al. [5] addressed the issue of Taiwan’s cost containment strategies on pharmaceutical expenditures. The authors of that study also applied Box-Tiao intervention analysis to examine the level change (the immediate effect) after the implementation of drug price adjustments. They found that pharmaceutical expenditures on all PBS listed drugs significantly decreased after the introduction of price adjustments based on generic grouping (PA2001 and PA2003). Our study included a time trend variable in the model, which enabled us to investigate the long-term effects of price adjustments. In fact, we found that the long-term trend in expenditures on overall renin-angiotensin drugs increased after PA2001 and PA2007.

Exploring increases in expenditures on overall renin-angiotensin drugs, we found that annual incident renin-angiotensin drug users declined over time, and no significant trend increases were found in the utilization of renin-angiotensin drugs over the study period. We also found that annual incident ARB users and annual prevalent ARB users increased over time. In particular, the annual prevalent ARB users always exceeded the number of cumulative incident ARB users, indicating that annual prevalent ARB users were not only from the category of cumulative incident ARB users, but also patients who were ever treated with ACEIs. These findings suggest that the increase in expenditures on renin-angiotensin drugs throughout this study primarily resulted from increases in the number of incident ARB users and potential switching from ACEIs to ARBs, but not from changes in the use of overall renin-angiotensin drugs.

Although the health care systems in Canada and European countries are different from that in Taiwan, and the reference pricing (RP) scheme [18] adopted in these countries control cost from the demand side (patients) but not from the providers (physicians) side, some of the studies conducted reveal that the RP scheme did not have any long-term effects. Evidence from the Netherlands showed that, after the implementation of the RP scheme, the cost of drugs covered by the RP scheme increased less than predicted, but the cost of drugs outside of the RP scheme has increased annually [19]. In addition, evidence from Germany and Hungary showed that pharmaceutical expenditures are still not well controlled because of volume growth that occurred after the implementation of the RP scheme [20,21].

Previous studies have reported that the use of cheaper, generic drugs may lead to cost savings, but clinical concerns regarding patient safety and therapeutic effectiveness related to treatment discontinuation have also been raised [22-25]. Our study demonstrated that prescription switching was from cheaper drugs to more expensive agents, and our patients, with a complexity of clinical conditions, were more likely to be treated with both drugs (subsequent or concurrent use of ACEI and ARB) than ACEIs alone. It is noteworthy that a difference between ARBs and ACEIs may be the persistent coughing caused by ACEIs. Patients who cannot tolerate ACEIs often switch to ARBs. No differences in the clinical recommendations for ARBs and ACEIs were noted during the study period [7]. Further analyses are warranted to compare the effectiveness and economic outcome for patients treated with ACEIs, those treated with ARBs, and those who switched from ACEIs to ARBs.

This study had several limitations. First, the baseline disease severity which led to initiation of treatment with ACEIs or ARBs was not compared, and we were unable to distinguish the clinical appropriateness of stopping or switching drugs for individual patients. The assessment of clinical conditions using ICD9-CM codes is likely to minimize most, but not all, of the potential bias. Second, there was no control group in this study, because price adjustments were implemented nationwide concurrently. However, the time points in the pre-intervention period served as a control group for the post-intervention period in the intervention analysis. Thus, issues regarding internal validity (such as history and maturation) were taken into consideration [14]. Finally, because the time period between price adjustments is quite short, incorporating each price adjustment’s level change and trend change into the intervention model would show severe multicollinearity, and it would be difficult to achieve significance for the collinearity parameters. That is why some previous studies only examined the level change, but not the trend change. However, since the trend change represents the long-term effect of policy interventions, its implication is greater than what the level change can explain and so it cannot be ignored. Therefore, we adopted a parsimonious model instead of a full model [14], keeping only the significant predictors selected by a backward elimination procedure and collinearity diagnostics. In this way, the long-term effect of the price adjustments can be examined and the multicollinearity problem can be avoided.

## Conclusions

The implementation of Taiwan’s stepwise price adjustments, with different adjustment strategies applied to patented and off-patent drugs, has achieved cost savings for off-patent ACEIs, but not for patented ACEI, ARBs and overall renin-angiotensin drugs in the long term. Increases in incident ARB users and possible switching from ACEIs to ARBs have emerged. These results indicate that policy makers in Taiwan should reconsider the appropriateness of the current adjustment strategies applied to patented and off-patent products, since they result in a difference in the profit margin that physicians obtain from these two classes of drugs and affect physicians’ prescribing decisions.

## Competing interests

The authors declare that they have no competing interests.

## Authors’ contributions

SHH conceived of the study, participated in its design, conducted data management and statistical analysis, interpreted the results and drafted the manuscript. CNH participated in the study design, statistical analysis, interpretation of the results, and drafting of the manuscript. SHY participated in the statistical analysis, interpretation of the results, and drafting of the manuscript. TMC conceived of the study, and participated in its design and coordination, interpretation of the results, as well as drafting of the manuscript. All authors read and approved the final manuscript.

## Supplementary Material

Additional file 1 **The principles of Taiwan’s price adjustments based on the market price and volume surveys, as well as generic grouping.** This file describes the background of Taiwan’s pharmaceutical benefit scheme (PBS) and delineates in detail the process of stepwise price adjustment. It also provides an example to show how lowering the reimbursement price decreases the profit for physicians. Click here for file
