# Supplementary material for: Impact of drug price adjustments on utilization of and expenditures on angiotensin-converting enzyme inhibitors and angiotensin receptor blockers in Taiwan
Source: BMC Public Health. 2012 Apr 20;12:288. doi: 10.1186/1471-2458-12-288 (PMC3464938; doi:10.1186/1471-2458-12-288)
Supplement: Additional file 1 — The principles of Taiwan’s price adjustments based on the market price and volume surveys, as well as generic grouping. This file describes the background of Taiwan’s pharmaceutical benefit scheme (PBS) and delineates in detail the process of stepwise price adjustment. It also provides an example to show how lowering the reimbursement price decreases the profit for physicians. [file 1471-2458-12-288-S1.pdf]

The principles of Taiwan's price adjustments based on market price and volume survey as well as generic grouping

Principles:

Initially, the principles of approval of reimbursement prices (known as the new drug entry price) in Taiwan were based on drug prices in the top 10 industrialized countries (i.e., England, Germany, Japan, Switzerland, USA, Belgium, Australia, France, Sweden, and Canada). For drugs with identical ingredients and dosage form, the reimbursement prices were highest in patented original branded products and under-surveillanced, off-patent original branded products (prices should be as ≤ the median price in the top 10 industrialized countries), followed by non-under-surveillanced, off-patent original branded products (prices should be as ≤ 85% of the median price in the top 10 industrialized countries), bioavailability/bioequivalent (BA/BE) generics (drugs that have performed BA/BE study; prices should be as ≤ the original branded products), and non-BA/BE generics (prices should be as ≤ 80% of the original branded products and should be as ≤ the BA/BE generics)[1].

Subsequently, the reimbursement prices for products listed in Pharmaceutical Benefit Scheme (PBS) established by the Bureau of National Health Insurance (BNHI) were adjusted gradually in the later years. The earlier stages of price adjustments (1996–1997) were based on the prices of international products or existing products (inter-brand comparison). In 2000, the BNHI in Taiwan began to conduct market price and volume survey before each price adjustment. At the price adjustment implemented on April 1, 2000 (PA2000), the BNHI adjusted each individual

product's reimbursement price according to the following equation [2]:

1. If WAP ≥ (1–r) × P_{old} then reimbursement price is not adjusted;

2. If WAP < (1–r) × P_{old} then reimbursement price is adjusted by the following:

$$P_{new} = WAP + P_{old} \times r$$

P_{old}, reimbursement price for each individual product before adjusting

P_{new}, new reimbursement price for each individual product after adjusting

r, the accessible profit margin for physicians imposed by BNHI (see table A and figure A)

WAP, weighted average market trading price for each individual product calculated from "n" physicians' purchasing volume and price obtained from the market survey

$$WAP = \frac{\sum_{i=1}^{n} volume_i \times price_i}{\sum_{i=1}^{n} volume_i}$$

Since the price adjustment that was implemented on April 1, 2001 (PA2001), the reimbursement prices of patented and off-patent products have been adjusted by the BNHI according to different strategies as follows:

1. For patented drugs (including some drugs qualified as 7-year market exclusivity in Taiwan), the individual drug's price was still adjusted based on the above equation, and the "r-zone" was shown in Table A.
2. For off-patent drugs (drugs without intellectual right argument or far-year drugs), the principle of generic grouping was applied first to group drugs with identical active ingredients, content, and strength. Subsequently, products in the same group

were further classified into three classes: (a) original brands, generics or biological products for which bioequivalent/bioavailability (BA/BE) tests have been performed; (b) BA/BE products outside the first class; and (c) drugs for which BA/BE tests have not been conducted. For products within the same group and class, a group weighted average market-trading price (GWAP) was calculated from all of these products' volume and price purchased by physicians obtained from market survey. The price of off-patent drug was adjusted also according to the above equation with modification in some parts:

(1) WAP was replaced by GWAP in the above equation unless GWAP was larger than WAP.

(2) Reimbursement price for class (a) drugs (i.e., original branded off-patent drugs) was calculated using 1.05 times the value of GWAP in the above equation.

(3) The "r-zone" is shown in Table A.

Table A. The r-zone set at each price adjustment

Price adjustments	r-zone[1] for different products	
	Patented products	Off-patent products
PA2000	30%	30%
PA2001	16%	16%
PA2003	16%	16%
PA2004	16%	16%
PA2005	16%	16%
PA2006	15%	6%
PA2007	15%	6%

[1]r-zone, a reasonable zone above the weighted average market trading price imposed by BNHI to allow an accessible profit margin for physicians
PA, price adjustment
PA2000, price adjustment implemented on April 1, 2000; PA2001, price adjustment implemented on April 1, 2001; PA2003, price adjustment implemented on March 1, 2003; PA2004, price adjustment implemented on November 1, 2004; PA2005, price adjustment implemented on September 1, 2005; PA2006, price adjustment implemented on November 1, 2006; PA2007, price adjustment implemented on September 1, 2007.

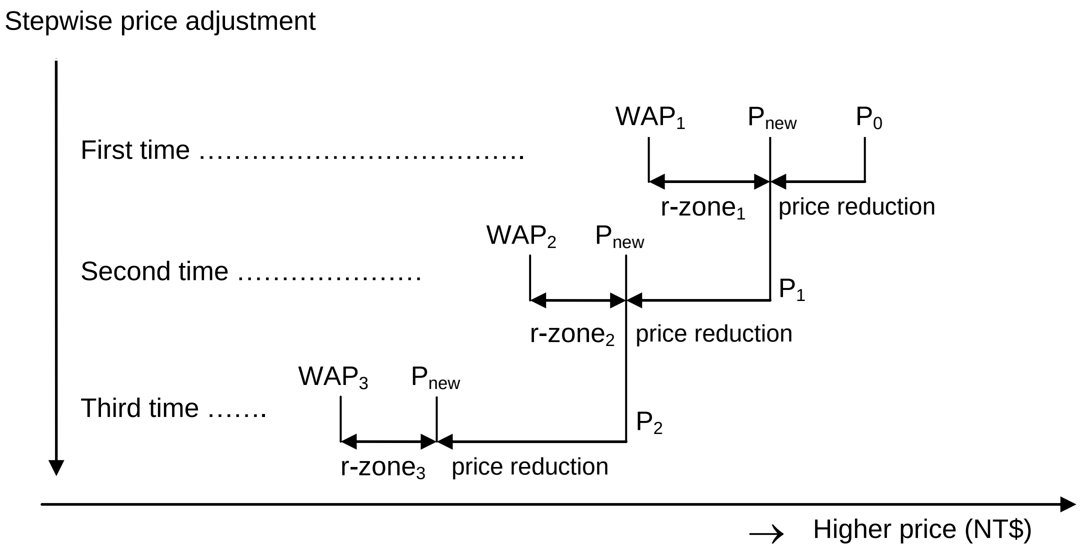

Figure A. The relationship between reimbursement price, market trading price and r-zone
At the price adjustment implemented in 2000, all of the individual products' prices were adjusted according to WAP. Since the price adjustment implemented in 2001, off-patent products' prices were adjusted according to the same group of drugs' group weighted average market trading price (GWAP) instead of individual drug's weighted average market trading price (WAP). $WAP_{1\ to\ 3}$, the weighted average market trading price calculated from market survey before 1st to 3rd time price adjustment; P_0, new drug entry price; $P_{1\ to\ 3}$, the reimbursement price after 1st to 3rd time price adjustment; P_{new}, the reimbursement price after each price adjustment; r-zone$_{1\ to\ 3}$, accessible profit margin for physicians set at 1st to 3rd price adjustment.

Hypothesized example:

<u>Background information for the example drug – Enalapril:</u>

Enalapril was an angiotensin-converting enzyme inhibitor (ACEI) defined as patented drug by the BNHI before PA2006. Therefore, the price adjustment for this drug was according to its individual product's weighted average market trading price (WAP), and the r-zone for this drug was set at 30% in PA2000 and 16% in PA2001 as well as in PA2003. Since PA2006, it became an off-patent product. Thus, the price adjustment for all brands of this drug was according to the same group of drugs' group weighted average market trading price (GWAP), and the r-zone for this drug

was set at 6%. Table B shows how lowering the reimbursement price decreases the profit for physicians.

Table B. The change of reimbursement price and physicians' profit of Enalapril before and after each price adjustment

	PA2000	PA2001	PA2003	PA2006	PA2007
r-zone[1] (%)	30.0	16.0	16.0	6.0	6.0
WAP or GWAP[2] (NT$)	14	14	13.2	8.1	6.5
P_{old} (NT$)	17.2	17.2	16.8	15.9	9.1
P_{new} (NT$)	17.2	16.8	15.9	9.1	7.0
$P_{reduction}$ (NT$)	0	0.4	0.9	6.8	2.1
$Profit_{old}$ (NT$)	3.2	3.2	3.6	7.8	2.6
$Profit_{new}$ (NT$)	3.2	2.8	2.7	1.0	0.5
$Profit_{reduction}$ (NT$)	0	0.4	0.9	6.8	2.1

[1]r-zone, a reasonable zone above the weighted average market trading price imposed by BNHI to allow an accessible profit margin for physicians
[2]Before PA2006, the price adjustment of Enalapril was according to WAP. Since PA2006, Enalapril turned to off-patent product and the price adjustment of Enalapril was according to GWAP.
PA, price adjustment; WAP, weighted average market trading price; GWAP, group weighted average market trading price; NT$, New Taiwan Dollars; at an exchange rate of NT$30.39 to US$1 on June 30, 2008; P_{old}, reimbursement price before each price adjustment; P_{new}, reimbursement price after each price adjustment; $P_{reduction}$, the reduction from P_{old} to P_{new}; $Profit_{old}$, the profit for physicians before each price adjustment (i.e., P_{old} minus WAP or P_{old} minus GWAP); $Profit_{new}$, the profit for physicians after each price adjustment (i.e., P_{new} minus WAP or P_{new} minus GWAP); $Profit_{reduction}$, the reduction from $Profit_{old}$ to $Profit_{new}$; PA2000, price adjustment implemented on April 1, 2000; PA2001, price adjustment implemented on April 1, 2001; PA2003, price adjustment implemented on March 1, 2003; PA2006, price adjustment implemented on November 1, 2006; PA2007, price adjustment implemented on September 1, 2007.

This hypothesized example displays that throughout the stepwise price adjustments, the difference between the reimbursement price and the market trading price (i.e., profit for physicians) narrowed. It is also important to find that when the patented Enalapril turned to an off-patent Enalapril (at the time of PA2006 implementation), the reimbursement price of this drug is adjusted according to a lower GWAP as well as a smaller r-zone compared with the WAP and the r-zone before PA2006, and therefore, the reduction in reimbursement price is larger and the profit margin for physicians is significantly squeezed.

References:

1. **Principles on Drug Reimbursement Price Approval of National Health Insurance**

   [http://www.nhi.gov.tw/resource/Webdata/Attach_4161_1_chi65-3.doc]

2. **Guidelines of price adjustment for National Health Insurance reimbursed drugs**

   [http://www.nhi.gov.tw/english/webdata.asp?menu=11&menu_id=295&webdata_id=2444]
